# The role of intraoperative swab during appendectomy in patients with uncomplicated and complicated appendicitis

**DOI:** 10.1007/s00384-023-04566-8

**Published:** 2023-11-22

**Authors:** Bruno Leonardo Bancke Laverde, Matthias Maak, Melanie Langheinrich, Stephan Kersting, Axel Denz, Christian Krautz, Georg F. Weber, Robert Grützmann, Maximilian Brunner

**Affiliations:** 1 Department of General and Visceral Surgery, Friedrich-Alexander-University Erlangen-Nürnberg (FAU), Krankenhausstraße 12, 91054 Erlangen, Germany; 2 Department of General, Visceral, Thoracic and Vascular Surgery, University Greifswald, Ferdinand-Sauerbruch-Straße, 17475 Greifswald, Germany

**Keywords:** Uncomplicated appendicitis, Complicated appendicitis, Appendectomy, Intraoperative swab, Bacterial isolation

## Abstract

**Introduction:**

Bacteria play an important role not only in pathogenesis of appendicitis but also in the postoperative course of patients. However, the usefulness of an intraoperative swab during appendectomy is controversial. The primary aim of this study was to investigate the impact of intraoperative swab during appendectomy on the postoperative outcome in patients with uncomplicated and complicated appendicitis.

**Methods:**

A retrospective analysis was conducted on a consecutive series of 1570 adult patients who underwent appendectomy for acute appendicitis at the University Hospital Erlangen between 2010 and 2020. Data regarding the intraoperative swab were collected and analyzed for the entire cohort as well as for patients with uncomplicated and complicated appendicitis.

**Results:**

An intraoperative swab was taken in 29% of the cohort. The bacterial isolation rate in the obtained intraoperative swabs was 51%, with a significantly higher rate observed in patients with complicated appendicitis compared to those with uncomplicated appendicitis (79% vs. 35%, *p* < 0.001). The presence of a positive swab was significantly associated with worse postoperative outcomes, including higher morbidity, increased need for re-surgery, and longer hospital stay, when compared to patients without a swab or with a negative swab. A positive swab was an independent risk factor for postoperative morbidity (OR 9.9 (95% CI 1.2–81.9), *p* = 0.034) and the need for adjustment of postoperative antibiotic therapy (OR 8.8 (95% CI 1.1–72.5), *p* = 0.043). However, a positive swab resulted in postoperative antibiotic therapy adjustment in only 8% of the patients with bacterial isolation in the swab.

**Conclusion:**

The analysis of swab samples obtained during appendectomy for acute appendicitis can help identify patients at a higher risk of a worse postoperative outcome. However, the frequency of antibiotic regime changes based on the swab analysis is low.

## Introduction

Acute appendicitis is a prevalent surgical emergency, with a lifetime incidence estimated to range from 7 to 14% [1]. Traditionally, bacterial proliferation resulting from appendix lumen obstruction has been considered the main cause of appendiceal inflammation [2–4]. However, emerging evidence suggests alternative pathogenetic mechanisms, including influence of the colonic microbiome and the extent of appendicitis (uncomplicated versus complicated) [2, 5]. However, bacterial infection remains a decisive factor not only in pathogenesis but also in the postoperative course.

Surgery is the standard treatment for acute appendicitis, with laparoscopic approach being favored due to its well-documented benefits, including reduced postoperative pain, lower complication rates, shorter hospital stays, and faster return to oral intake [6–9]. Nevertheless, despite a successful appendectomy, some patients experience postoperative complications, such as surgical site infections or intraabdominal abscesses.

Obtaining intraoperative fluid samples for microbiological culture is a common practice aimed at identifying bacteria and facilitating targeted antibiotic therapy. However, some authors argue that peritoneal swabs are an unnecessary use of resources and do not impact the management of postoperative complications [10–13].

The primary objective of this study was to examine the role of intraoperative swab during appendectomy in influencing the postoperative course of patients with uncomplicated and complicated appendicitis.

## Methods

This retrospective cohort study included a total of 1570 consecutive patients aged 18 years or older who underwent appendectomy for acute appendicitis at the Surgery Department of the University Hospital between 2010 and 2020. Patients who underwent appendectomy during other surgical procedures or those without intraoperative and/or histopathological diagnosis of acute appendicitis were excluded from the study.

In addition to data about the intraoperative swab and its microbiological analysis, the collected data for analysis included patient demographics, preoperative blood tests and radiological findings, intraoperative findings, surgical approach, surgery duration, and presence of malignancy. The collected data included inpatient records and, if applicable, follow-up visits. There was no structured follow-up. Data analysis was conducted for the entire patient cohort, as well as stratified according to patients with uncomplicated and complicated appendicitis. The primary objective was to assess the influence of intraoperative swabs and bacterial isolation on various outcome parameters (morbidity, infectious morbidity, major morbidity, re-surgery, and prolonged hospital stay). The secondary objective involved conducting univariate and multivariate risk factor analyses to identify predictive factors for bacterial isolation in intraoperative swabs, in-hospital postoperative morbidity, and the requirement for postoperative antibiotic therapy adjustments.

The study protocol was approved by the FAU University Ethical Commission (22–157-Br). The work has been reported in line with the STROCSS criteria [14].

### Definitions

Complicated appendicitis was defined as gangrenous or perforated appendicitis with or without abscess or peritonitis. Classification into uncomplicated and complicated cases was based on the intraoperative findings. Morbidity was assessed during the hospital stay and if readmission occurred and was classified according to the Clavien-Dindo classification [15], with any deviation from the ordinary postoperative course considered morbidity. Any complications that occurred outside of our clinic and were treated elsewhere could not be included due to a lack of information. Major morbidity was defined as morbidity of grade IIIa or higher. Surgical site infection and intraabdominal abscess were considered infectious morbidity. A hospital stay was considered prolonged if it exceeded 5 days.

### Surgical procedure and postoperative management

The choice of surgical approach for appendectomy was based on the surgeon’s preference. Initially, during the early years of the study period, the open approach was more frequently employed. However, in recent years, laparoscopic appendectomy accounted for over 95% of all cases. The decision to obtain an intraoperative swab as well as to continue the intraoperative single shot antibiotic prophylaxis as postoperative antibiotic therapy was at the discretion of the operating surgeon. The location of the intraoperative swab was determined by the surgeon based on the intraoperative findings and the presence of free fluid or a perityphlitic abscess. If the swab provided sufficient material for both tests, both culture and PCR were performed. Otherwise, only a culture was set up. For patients with an obtained intraoperative swab, the antibiotic therapy was adjusted based on the results of the swab culture. The standard first-line antibiotic therapy consisted of a combination of cefotaxime and metronidazole.

### Statistical analysis

Statistical analyses were performed using SPSS Statistics (Version 22.0, IBM). Ordinal and metric data were compared using the Mann–Whitney U test or Student t-test. The chi-square test was used for categorical data. Statistical significance was set at *p* < 0.05. Multivariate analysis was performed to identify predictive factors for bacterial isolation in obtained intraoperative swabs, postoperative in-hospital morbidity, and the need for adjustment of postoperative antibiotic therapy. Only parameters that showed a significant association with positive swab, morbidity, and change of postoperative antibiotic therapy in the univariate analysis were included in the multivariate analysis. The median was chosen as the cutoff for metric parameters in the multivariate analysis.

## Results

### Demographic data

Out of the 1570 patients included in the study (median age: 35 years; 48% female), 1174 had uncomplicated appendicitis while 396 had complicated appendicitis. Patients with complicated appendicitis were significantly older (51 vs. 31 years, *p* < 0.001), had a higher BMI (24.2 vs. 25.8 kg/m^2^, *p* < 0.001), worse ASA score (*p* < 0.001), a higher prevalence of diabetes (8 vs. 3%, *p* > 0.001), higher CRP value (117 vs. 22 mg/l, *p* < 0.001), and a higher prevalence of intraabdominal fluid on preoperative sonography (54 vs. 31%, *p* < 0.001). Additionally, patients with complicated appendicitis had a higher prevalence of open and converted appendectomy (*p* < 0.001), longer surgery duration (76 vs. 59 min, *p* < 0.001), and a greater need for cecum resection (13 vs. 2%, *p* < 0.001) (Table 1).

**Table 1:** Demographic data

	**All patients**	**Uncomplicated appendicits**	**Complicated appendicitis**	***p*-value**
Number, *n* (%)	1570	1174 (75)	396 (25)	
Age (years), median (IQR)	35 (26)	31 (22)	51 (28)	** < 0.001**
Gender, *n* (%)				0.163
Female Male	747 (48) 823 (52)	571 (49) 603 (51)	176 (44) 220 (56)	
BMI* (kg/m^2^), median (IQR)	24.5 (5.9)	24.2 (5.7)	25.8 (6.1)	** < 0.001**
ASA*, *n* (%)				** < 0.001**
I II III IV	872 (60) 479 (33) 92 (6) 5 (0)	724 (67) 324 (30) 35 (3) 0 (0)	148 (41) 155 (43) 57 (16) 5 (1)	
Diabetes, *n* (%)	69 (4)	36 (3)	33 (8)	** < 0.001**
Preoperative diagnostics				
CRP (mg/l), median (IQR) Intraabdominal fluid, *n* (%)	38 (87) 579 (37)	22 (59) 366 (31)	117 (136) 213 (54)	** < 0.001** ** < 0.001**
Intraoperative findings, *n* (%)				
Perforation Necrosis or gangrene Perithyphilitic abscess	340 (22) 129 (8) 161 (10)	0 (0) 0 (0) 0 (0)	340 (86) 129 (33) 161 (41)	** < 0.001** ** < 0.001** ** < 0.001**
Surgical approach, *n* (%)				** < 0.001**
Laparoscopic Open Conversion	1153 (74) 350 (22) 67 (4)	926 (79) 233 (20) 15 (1)	227 (57) 117 (30) 52 (13)	
Duration of surgery (min), median (IQR)	62 (32)	59 (27)	76 (41)	** < 0.001**
Need of cecum resection, *n* (%)	69 (4)	18 (2)	51 (13)	** < 0.001**
Swab taken, *n* (%)	457 (29)	292 (25)	165 (42)	** < 0.001**
Bacterial isolation in swab**, *n* (%)	233 (51)	102 (35)	131 (79)	** < 0.001**

^*^ missing data > *n* = 1137 (BMI) and 1448 (ASA); ** only patients with swab taken (*n* = 457)

Intraabdominal swabs were obtained from 457 patients (29%), with a higher frequency observed in patients with complicated appendicitis (42 vs. 25%, *p* < 0.001). Among the 457 collected intraoperative swabs, bacteria could be isolated in 233 cases (51%), with a higher isolation rate in patients with complicated appendicitis (79 vs. 35%, *p* < 0.001) (Table 1).

Regarding the postoperative outcome, morbidity, infectious morbidity, major morbidity, need of re-surgery, or prolonged hospital stay occurred in 6%, 2%, 3%, 1%, and 13%, respectively (Table 2).

**Table 2:** Bacteria analysis in all patients with appendectomy for acute appendicitis (*n* = 1570)

	**Number of patients**	**Morbidity**		**Infectious morbidity**		**Major morbidity**		**Re-surgery**		**Prolonged hospital stay (> 5 POD)**	
		***n* (%)**	***p***	***n* (%)**	***p***	***n* (%)**	***p***	***n* (%)**	***p***	***n* (%)**	***p***
All patients	1570	99 (6)		32 (2)		50 (3)		21 (1)		209 (13)	
Number of bacteria			** < 0.001**		**0.035**		**0.001**		** < 0.001**		** < 0.001**
No swab 0 1 2 ≥ 3	1113 (71) 224 (14) 78 (5) 68 (4) 87 (6)	65 (6) 3 (1) 5 (6) 10 (15) 16 (18)		24 (2) 1 (0) 0 (0) 2 (3) 5 (6)		31 (3) 2 (1) 3 (4) 6 (9) 8 (9)		9 (1) 2 (1) 2 (3) 2 (3) 6 (7)		132 (12) 5 (2) 15 (19) 17 (25) 40 (46)	
No swab vs. swab			0.253		0.697		0.205		**0.007**		** < 0.001**
Sterile swab vs. positive swab			** < 0.001**		**0.037**		** < 0.001**		**0.037**		** < 0.001**
No swab vs. sterile swab			**0.007**		0.104		0.103		1.000		** < 0.001**
No swab vs. positive swab			** < 0.001**		0.469		**0.002**		** < 0.001**		** < 0.001**
Patients with bacterial isolation in swab	233	31 (13)		7 (3)		17 (7)		10 (4)		102 (44)	
Kind of bacteria*											
* E. coli* * Bacterioides spp.* * Streptococcus spp.* * Pseudomonas spp.* Others	128 (55) 80 (34) 55 (24) 36 (15) 146 (63)	24 (19) 13 (17) 7 (13) 7 (19) 17 (12)	**0.011** 0.415 1.000 0.283 0.425	7 (6) 1 (1) 3 (6) 1 (3) 5 (3)	**0.017** 0.428 0.360 1.000 0.715	11 (9) 6 (8) 4 (7) 3 (8) 10 (7)	0.456 1.000 1.000 1.000 0.797	6 (5) 3 (4) 3 (6) 2 (6) 7 (5)	0.761 1.000 0.704 1.000 0.747	74 (58) 40 (51) 30 (55) 22 (61) 61 (42)	** < 0.001** 0.163 0.087 **0.028** 0.495

bold = significant

*POD* postoperative days, *spp.*, species

* only patients with bacterial isolation in swab

### Bacteria analysis

In comparison to patients with no intraoperative swab or a sterile intraoperative swab, those with a positive intraoperative swab exhibited higher rates of morbidity (13 vs. 6%, *p* < 0.001; 13 vs. 1%, *p* < 0.001), major morbidity (7 vs. 3%, *p* = 0.002; 7 vs. 1%, *p* < 0.001), re-surgery (4 vs. 1%, *p* < 0.002; 4 vs. 1%, *p* = 0.037), and prolonged hospital stay (31 vs. 12%, *p* < 0.001; 31 vs. 2%, *p* < 0.001). When comparing patients with no swab and those with a sterile swab, the sterile swab group showed only a favorable outcome in terms of morbidity and the rate of prolonged hospital stay (*p* = 0.007 and *p* < 0.001, respectively). As for infectious morbidity, a significant difference was observed only between the sterile swab and positive swab groups (0 vs. 3%, *p* = 0.037) (Table 2). The number of bacteria detected correlated positively with the prevalence of morbidity, infectious morbidity, major morbidity, reoperations, and the need for prolonged hospital stays (Table 2).

Stratified to the extent of appendicitis, in uncomplicated appendicitis again a positive swab was associated with a worse outcome (morbidity, major morbidity, re-surgery, and prolonged hospital stay) compared to patients with no intraoperative swab (*p* = 0.013, *p* = 0.004, *p* = 0.003, *p* < 0.001, respectively) and with a sterile swab (*p* = 0.001, *p* = 0.002, *p* = 0.014, *p* < 0.001, respectively). In patients with complicated appendicitis, only a sterile swab showed a protective effect against prolonged hospital stay compared to those with no intraoperative swab (*p* < 0.001) and with a positive swab (*p* < 0.001) (Table 3).

**Table 3:** Bacteria analysis in all patients with appendectomy for acute appendicitis stratified to uncomplicated (*n* = 1174) and complicated appendicitis (*n* = 396)

	**Number of patients**	**Morbidity**		**Infectious morbidity**		**Major morbidity**		**Re-surgery**		**Prolonged hospital stay (> 5 POD)**	
		***n* (%)**	***p***	***n* (%)**	***p***	***n* (%)**	***p***	***n* (%)**	***p***	***n* (%)**	***p***
**Patients with uncompl. app**	1174	33 (2)		6 (1)		16 (1)		7 (1)		57 (5)	
Number of bacteria			**0.006**		0.231		**0.003**		**0.018**		** < 0.001**
No swab 0 1 2 ≥ 3	882 (75) 190 (16) 51 (4) 28 (2) 23 (2)	24 (3) 1 (1) 2 (4) 3 (11) 3 (13)		5 (1) 0 (0) 0 (0) 1 (4) 0 (0)		10 (1) 0 (0) 2 (4) 3 (11) 1 (4)		3 (0) 0 (0) 2 (4) 1 (4) 1 (4)		39 (4) 2 (1) 6 (12) 3 (11) 7 (30)	
No swab vs. swab			0.838		1.000		0.248		0.069		0.270
Sterile swab vs. positive swab			**0.001**		0.349		**0.002**		**0.014**		** < 0.001**
No swab vs. sterile swab			0.106		0.593		0.224		0.639		**0.034**
No swab vs. positive swab			**0.013**		1.000		**0.004**		**0.003**		** < 0.001**
**Patients with compl. app**	396	66 (17)		26 (7)		34 (9)		14 (4)		152 (38)	
Number of bacteria			0.373		0.290		0.799		0.199		**0.001**
No swab 0 1 2 ≥ 3	231 (58) 34 (9) 27 (7) 40 (10) 64 (16)	41 (18) 2 (6) 3 (11) 7 (18) 13 (20)		19 (8) 1 (3) 0 (0) 1 (3) 5 (8)		21 (9) 2 (6) 1 (4) 3 (8) 7 (11)		6 (3) 2 (6) 0 (0) 1 (3) 5 (8)		93 (40) 3 (9) 9 (33) 14 (35) 33 (52)	
No swab vs. swab			0.585		0.150		0.719		0.275		0.402
Sterile swab vs. positive swab			0.091		1.000		0.740		1.000		** < 0.001**
No swab vs. sterile swab			0.080		0.337		0.749		0.602		** < 0.001**
No swab vs. positive swab			1.000		0.205		0.851		0.364		0.658

bold = significant

*POD*, postoperative days; *uncompl.*, uncomplicated; *app.*, appendicitis; *compl.*, complicated

Considering bacteria species, especially two bacteria, had an impact on postoperative outcomes: *Escherichia coli* was associated with a higher morbidity (19 vs. 13%, *p* = 0.011) and infectious morbidity (6 vs. 3%, *p* = 0.017) and with a higher rate of prolonged hospital stay (58 vs. 44%, *p* < 0.001). The second bacteria were *Pseudomonas spp*., which was associated with a higher rate of prolonged hospital stay (61 vs. 44%, *p* = 0.028) (Table 2).

### Risk factors for positive swab after appendectomy

In the univariate analysis, eight risk factors for a positive swab were identified: older age (46 vs. 31 years, *p* < 0.001), higher BMI (24.8 vs. 24.1 kg/m^2^, *p* = 0.018), worse ASA score (*p* < 0.001), higher prevalence of diabetes (16 vs. 3%, *p* = 0.004), higher preoperative CRP value (92 vs. 33 mg/l, *p* < 0.001), higher intraoperative prevalence of perforation (49 vs. 13%, *p* < 0.001), of necrosis or gangrene (15 vs. 6%, *p* = 0.001), and of perityphlitic abscess (25 vs. 5%, *p* < 0.001) (Table 4). A multivariate analysis revealed intraoperative presence of perforation (OR 2.7, CI 1.5–5.2, *p* = 0.002) and of abscess (OR 3.8, CI 1.4–10.1, *p* = 0.008) as independent risk factors for bacterial isolation in the intraoperative swab (Table 5).

**Table 4:** Risk factors for positive swab and morbidity in patients undergoing appendectomy with intraoperatively taken swab for acute appendicitis (*n* = 457)

	**Sterile swab**	**Positive swab**	***p*-value**	**No morbidity**	**Morbidity**	***p*-value**	**No postop. AB change**	**Postop. AB change**	***p*-value**
Number, *n* (%)	224	233		423	34		437	20	
Age (years), median (IQR)	31 (24)	46 (28)	** < 0.001**	36 (27)	54 (25)	** < 0.001**	37 (27)	33 (34)	0.075
Gender, *n* (%)			0.224			1.000			1.000
Female Male	111 (50) 113 (50)	102 (44) 131 (56)		197 (47) 226 (53)	16 (47) 18 (53)		204 (47) 233 (53)	9 (45) 11 (55)	
BMI* (kg/m^2^), median (IQR)	24.1 (5.2)	24.8 (5.8)	**0.018**	24.5 (5.4)	27.4 (8.0)	**0.026**	24.1 (4.4)	27.8 (5.9)	0.239
ASA*, *n* (%)			** < 0.001**			** < 0.001**			
I II III IV	140 (67) 62 (30) 7 (3) 0 (0)	96 (46) 93 (44) 22 (10) 0 (0)		227 (58) 140 (36) 22 (6) 0 (0)	9 (29) 15 (48) 7 (23) 0 (0)		226 (56) 148 (37) 27 (7) 0 (0)	10 853) 7 (37) 2 (11) 0 (0)	0.801
Diabetes, *n* (%)	3 (1)	16 (7)	**0.004**	13 (3)	6 (18)	**0.001**	17 (4)	2 (10)	0.199
Preoperative diagnostics									
CRP (mg/l), median (IQR) Intraabdominal fluid, *n* (%)	33 (67) 96 (43)	92 (136) 117 (50)	** < 0.001** 0.133	44 (100) 189 (45)	130 (91) 24 (71)	** < 0.001** **0.004**	40 (77) 203 (47)	147 (120) 10 (50)	** < 0.001** 0.821
Intraoperative findings, *n* (%)									
Perforation Necrosis or gangrene Perithyphilitic abscess	28 (13) 13 (6) 11 (5)	119 (49) 35 (15) 57 (25)	** < 0.001** **0.001** ** < 0.001**	119 (28) 43 (10) 55 (13)	23 (68) 5 (15) 13 (38)	** < 0.001** **0.561** ** < 0.001**	130 (30) 46 (11) 61 (14)	12 (60) 2 (10) 7 (35)	**0.006** 1.000 **0.019**
Surgical approach, *n* (%)			** < 0.001**			0.335			0.075
Laparoscopic Open Conversion	123 (55) 100 (45) 1 (0)	112 (48) 104 (45) 17 (7)		218 (52) 190 (45) 15 (4)	17 (50) 14 (41) 3 (9)		220 (50) 200 (46) 17 (4)	15 (75) 4 (20) 1 (5)	
Duration of surgery (min), median (IQR)	58 (28)	70 (39)	** < 0.001**	62 (33)	76 (41)	**0.006**	60 (34)	69 (24)	**0.010**
Need of cecum resection, *n* (%)	4 (2)	22 (9)	** < 0.001**	21 (5)	5 (15)	**0.035**	25 (6)	1 (5)	1.000
Positive swab, *n* (%)	-	-	-	202 (48)	31 (91)	** < 0.001**	214 (49)	19 (95)	** < 0.001**
Postoperative antibiotic therapy, *n* (%)	88 (39)	182 (78)	** < 0.001**	239 (57)	31 (91)	** < 0.001**			
Change of postoperative antibiotic therapy, *n* (%)	1 (0)	19 (8)	** < 0.001**	9 (2)	11 (32)	** < 0.001**			
Morbidity, *n* (%)	3 (1)	31 (13)	** < 0.001**				23 (5)	11 (55)	** < 0.001**

bold = significant

*AB*, antibiotic

* missing data > *n* = 336 (BMI) and 420 (ASA)

**Table 5:** Multivariate analysis regarding risk factors for bacterial isolation in swab and morbidity in patients undergoing appendectomy with intraoperatively taken swab for acute appendicitis (*n* = 457)

	**Bacterial isolation in swab**			**Morbidity**			**Change of antibiotic therapy**		
	OR	CI	*p*-value	OR	CI	*p*-value	OR	CI	*p*-value
Age (high vs. low)*	1.3	0.7–2.3	0.427	1.6	0.5–5.4	0.415	-	-	-
BMI (high vs. low)*	1.0	0.6–1.8	0.981	1.3	0.5–3.8	0.594	-	-	-
ASA (III/IV vs. I/II)	2.6	0.6–11.1	0.196	1.1	0.2–5.0	0.916	-	-	-
Diabetes (yes vs. no)	1.4	0.2–7.9	0.723	1.5	0.2–9.3	0.696	-	-	-
Preoperative CRP (high vs. low)*	1.7	1.0–3.1	0.061	2.6	0.6–10.0	0.181	2.3	0.6–9.3	0.231
Intraabdominal fluid in radiological diagnostic (yes vs. no)	-	-	-	2.4	0.8–7.4	0.127	-	-	-
Intraoperative perforation (yes vs. no)	**2.7**	**1.5–5.2**	**0.002**	2.7	0.8–8.9	0.106	1.2	0.4–3.9	0.705
Intraoperative necrosis or gangrene (yes vs. no)	2.0	0.8–5.1	0.132	-	-	-	-	-	-
Intraoperative abscess (yes vs. no)	**3.8**	**1.4–10.1**	**0.008**	1.3	0.4–3.8	0.670	1.1	0.3–3.4	0.928
Surgical approach (open vs. laparoscopic vs. conversion)				-	-	-	-	-	-
Duration of surgery (high vs. low)*				1.2	0.4–3.6	0.751	2.7	0.8–9.6	0.120
Coecum resection (yes vs. no)				2.3	0.5–9.8	0.279	-	-	-
Positive swab (yes vs. no)				**9.9**	**1.2–81.9**	**0.034**	**8.8**	**1.1–72.5**	**0.043**
Morbidity (yes vs. no)							**10.9**	**3.8–31.4**	** < 0.001**

bold = significant

^*^ cut-off = median (for age: 38 years, for BMI: 24.5 kg/m^2^, for CRP: 51 mg/l, duration of surgery: 64 min)

### Risk for morbidity with an intraoperatively taken swab after appendectomy

Eleven parameters were associated with a higher morbidity in univariate analysis: older age (54 vs. 36 years, *p* < 0.001), higher BMI (27,4 vs. 24,5 kg/m^2^, *p* = 0.026), worse ASA score (*p* < 0.001), higher prevalence of diabetes (18 vs. 3%, *p* < 0.001), higher preoperative CRP value (130 vs. 44 mg/l, *p* < 0.001), presence of intraabdominal fluid in radiological diagnostic (71 vs. 45% *p* = 0.004), higher intraoperative prevalence of perforation (68 vs. 28%, *p* < 0.001) and perityphlitic abscess (38 vs. 13%, *p* < 0.001), longer surgery duration (76 vs. 62 min, *p* = 0.006), higher rate of necessity of cecum resection (15 vs. 5%, *p* = 0.035), and a higher rate of positive intraoperative swabs (91 vs. 48%, *p* < 0.001) (Table 4). In multivariate analysis, only a positive swab (OR 9.9, CI 1.2–91.9, *p* = 0.034) independently increased the risk of postoperative morbidity (Table 5).

### Risk factors for the need for adjustment of postoperative antibiotic therapy

Twenty of the 457 patients (4%) with an intraoperatively obtained swab required an adjustment of their postoperative antibiotic therapy, with a significant higher rate in patients with a positive swab compared to those with a negative swab (8 vs. 0%, *p* > 0.001). Six parameters were associated with the need for adjustment of the postoperative antibiotic therapy in univariate analysis: higher preoperative CRP value (147 vs. 40 mg/l, *p* < 0.001), higher intraoperative prevalence of perforation (60 vs. 30%, *p* = 0.006) and perityphlitic abscess (35 vs. 14%, *p* = 0.019), longer surgery duration (69 vs. 60 min, *p* = 0.010), higher rate of positive intraoperative swabs (95 vs. 49%, *p* < 0.001), and a higher rate of postoperative morbidity (55 vs 5%, *p* < 0.001) (Table 4). The multivariate analysis determined a positive swab (OR 8.8, CI 1.1–72.5, *p* = 0.043) and the occurrence of postoperative morbidity (OR 10.9, CI 3.8–31.4, *p* < 0.001) as independent risk factors for the need for adjustment of the postoperative antibiotic therapy (Table 5).

## Discussion

There is an ongoing debate about the usefulness of obtaining intraoperative swabs during an appendectomy. The main argument in favor is the ability to perform targeted antibiotic therapy during the postoperative period. Additionally, taking an intraoperative swab is quick, easily feasible, and incurs minimal costs per patient. Counterarguments include the infrequent consequential impact of obtaining an intraoperative swab, the inability to reduce the rate of intraabdominal abscesses through swab collection and antibiotic adjustment, and the overall relevant costs involved.

In our cohort, the rate of bacteria detection in intraoperative swabs was 51%, which falls within the range of positive cultures reported for acute appendicitis in the literature [11–13, 16]. The rate of bacteria detection varied significantly depending on the type of appendicitis (uncomplicated versus complicated), reaching 79% in patients with complicated appendicitis, which is consistent with a recent analysis [10]. In literature, besides the status of appendiceal inflammation (uncomplicated versus complicated), the collection technique is also mentioned to influence the detection rate [11–13]. However, this factor was not investigated in our analysis. Our study could identify a sonographically thicker appendix, intraoperative presence of perforation, and/or of abscess as independent predictive factors for bacterial isolation in the intraoperative swab.

According to the literature, anaerobic gram-negative bacteria have a higher prevalence in peritoneal fluid, followed by gram-positive bacteria, and anaerobic bacteria have the lowest prevalence [17]. Specifically, *Escherichia coli*, *Bacteroides fragilis*, *Pseudomonas aeruginosa*, *Proteus mirabilis*, and *Citrobacter freudii* have been reported to be commonly isolated in intraoperative swabs [3, 17, 18]. Our series demonstrated a higher incidence of *E. coli*, *Bacteroides* spp., *Streptococcus* spp., and *Pseudomonas* spp., which is consistent with previous data [10, 16]. In our study, an association with worse outcomes (morbidity, infectious morbidity, and prolonged hospital stay) was particularly observed for *E. coli*.

Our results showed that intraoperative swab analyses were associated with the postoperative course. Especially a positive intraoperative swab could be detected as an important parameter to identify patients with a higher risk of morbidity, major morbidity, re-surgery, and the need of a prolonged hospital stay in the whole cohort as well as in patients with uncomplicated appendicitis. In contrast, in patients with complicated appendicitis, a sterile intraoperative swab may be a protective parameter against a worse outcome, indicated in our data by the significantly lower rates of prolonged hospital stay. However, missing significance regarding the other outcome parameters in complicated appendicitis could be due to the smaller number of patients. In multivariate analysis, a positive swab could be identified as an independent risk factor for morbidity highlighting its significant predictive value. In summary, obtaining an intraoperative swab could help to identify high-risk patients in the group of uncomplicated appendicitis and low-risk patients in the group of complicated appendicitis.

However, when considering the specific outcome parameter of infectious morbidity, a significant distinction was observed only between patients with a sterile intraoperative swab and those with a positive swab in the entire cohort. Therefore, our data support the findings of the study conducted by Pena et al., which did not show a decrease in the incidence of postoperative intra-abdominal abscesses through the use of intraoperative swabs and adjustment of antibiotic therapy compared to those without an intraoperative swab [10].

Taking into account the implications derived from the results of the intraoperative swab analysis, a positive swab was found to be independently associated with a higher likelihood of requiring an adjustment in postoperative antibiotic therapy. However, a change in antibiotic therapy was only observed in 4% of all patients who underwent an intraoperative swab. In one patient, the antibiotic therapy was escalated despite a sterile swab result. In 8% of patients with a positive swab result, an escalation of antibiotic treatment was required. Therefore, the need for adjustment of antibiotic therapy in our cohort was low and lower than reported in other studies. For example, in the aforementioned study by Pena, approximately 36% of patients required a change in the empiric antibiotic regimen due to bacterial resistance [10]. Another series by Cimpean et al. demonstrated resistance rates of 9.3% in the complicated appendicitis group and 16.8% in the uncomplicated group against the empiric antibiotics used [3]. A potential explanation for the low rate of antibiotic therapy adjustments in our cohort could be attributed to our consistent utilization of broad-spectrum antibiotics as part of our standard practice. Furthermore, in addition to the limited impact on postoperative antibiotic therapy, the precise benefit of adjusting antibiotic therapy cannot be accurately determined based on retrospective data. This is because the comparison group without adjustments is likely to have inherent biases, such as less inflammation or a lower degree of complication severity, or the primary antibiotic therapy may have already been effective against the pathogens. These factors introduce potential biases when comparing it with the group that received adjusted antibiotic therapy. Therefore, a randomized controlled study is necessary to get better evidence to this topic [19].

This study has several limitations, primarily stemming from its retrospective design. There is a possibility of selection bias, as the decision to obtain intraoperative swabs was based on the surgeon’s criteria, which may have been influenced by the severity of the disease. This could explain the higher rate of obtained intraoperative swabs in the complicated appendicitis group. Additionally, our study did not investigate the costs associated with intraoperative swabs, which are crucial in assessing their usefulness through a cost/benefit analysis. There are always additional potential biases that cannot be ruled out, particularly in statistical aspects such as the risk of mass significance or differences between the groups, including the surgical approach.

## Conclusion

Bacteria isolation in swab samples obtained during appendectomy for acute appendicitis is associated with a higher incidence of postoperative morbidity, re-surgery, and of prolonged hospital stay. Consequently, intraoperative swabs can be beneficial in identifying patients at an elevated risk of experiencing adverse postoperative outcomes. However, the frequency of therapeutic regime changes related to antibiotic treatment is low and the impact of adjusting antibiotics on subsequent outcomes remains unclear.

## Data Availability

All data have been included in the manuscript and the tables.
